# *Mycobacterium avium-intracellulare* infection presenting as a testicular mass in an immunocompromised patient: a case report

**DOI:** 10.4076/1757-1626-2-8975

**Published:** 2009-08-19

**Authors:** Ruth A Hartley

**Affiliations:** Department of Haematology, East Surrey HospitalCanada Avenue, Redhill, Surrey, RH1 5RH, England

## Abstract

Isolated mycobacterial infections of the genital tract are extremely rare. Here we present a 63-year-old gentleman who developed an isolated non-tuberculous mycobacterial mass in the inferior pole of his right testis, secondary to immunocompromise related to multiple autoimmune and haematological conditions and their treatments.

## Introduction

Compromised immunity, associated with autoimmune and haematological conditions and their treatments, causes an estimated increase of 3.69 infections per patient, with a mortality of 21% [1]. Autoimmune conditions are also linked to the development of lymphoma, with a 3.16 relative risk of lymphoma developing in patients with systemic lupus erythematosis (SLE) [2]. The crossover between these two conditions can be profound [3], and the diagnostic intricacies can become even more complex given the prevalence of atypical infections which can mimic relapse of either condition.

Of the atypical infections, tuberculous and non-tuberculous mycobacteria (NTM) are common pathogens, most frequently in the chest, but exceptionally affecting the genitourinary tract [4]. Here we present a patient with an isolated NTM infection of his right testis due to immunocompromise from SLE, recent lymphoma, and immunosuppressive drugs.

## Case presentation

Our patient was a 63-year-old retired gentleman, weighing 64 kilograms and 1.8 meters tall. He had no significant family history and drank only occasionally. At presentation he was trying to give up a 40 pack-year smoking habit. His medications were predominately analgesics but also a bisphosphonate and prednisolone 20 mg.

He had a history of SLE, haemolytic anaemia, recurrent idiopathic thrombocytopenia purpura and recent chemotherapy (6 months previously) for diffuse large B-cell lymphoma.

He presented to clinic with a tender, diffusely enlarged right testicle, and was also noted to be suffering from night sweats, a 7 kg weight loss, and splenomegaly (previously noted prior to treatment for lymphoma).

**Figure 1.**
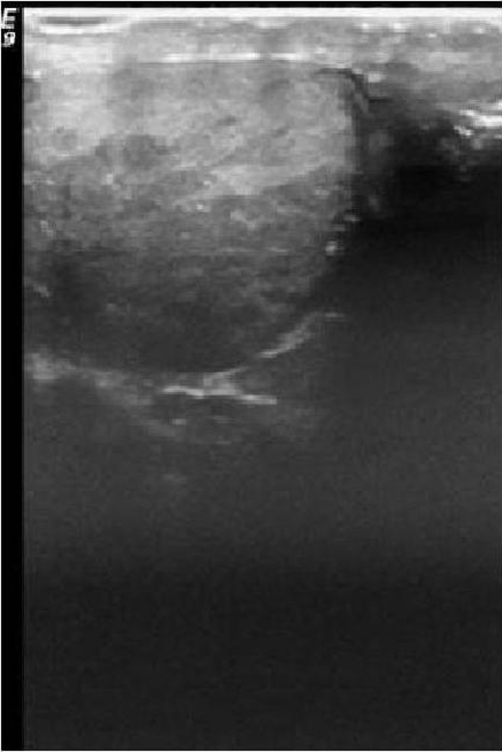
Ultrasound scan of the right testicle showing the non-homogenous abnormality inferiorly (white arrow).

Tumour markers were normal, but an ultrasound scan of his right testicle showed a non-homogenous abnormality inferiorly (Figure 1), in keeping with a recurrence of lymphoma or a primary malignancy [5].

Given his history and symptoms, he was urgently referred to the urologists who decided to proceed with a right orchidectomy.

Histology of the right testis showed a granulomatous orchiditis. A Ziehl-Neelson (ZN) stain showed a large number of acid fast bacilli (AFB) which, given the morphology and absence of caseation, was most consistent with a NTM infection. Unfortunately, no sample had been sent for culture, but given the histological findings and clinical presentation, this was considered most likely to be *Mycobacterium avium-intracellulare* (MAI) infection.

He recovered well post operatively, and, as a CT scan showed a right apical lung lesion, he was referred to the local respiratory physician for further investigation and management.

Bronchial washings of the right upper zone and left lingula were positive for AFB. He was commenced on conventional quadruple anti-tuberculous therapy pending formal identification of the AFB. The organism was subsequently confirmed as *MAI,* and therapy was switched according to the British Thoracic Society guidelines [6].

## Discussion

NTM infections in immunocompromised patients [7] are often associated with disseminated disease, with the chest typically as the primary focus of infection [8]. The genito-urinary system can become involved, as with tuberculous disease, but involvement of the testes without early epididymal changes, as occurred in this case, was previously considered as almost pathognomonic of a neoplastic rather than infectious process [1,9].

## Conclusion

Mycobacterial infections in immunocompromised patients must be excluded due to the importance in prognosis and treatment (early treatment of MAI results in remission rates of 67% even in the immunocompromised) [10]. In this gentleman, his extreme immunocompromised state made him the ideal candidate for opportunistic infections, resulting in the situation where unusual presentation of an infection mimicked a recurrence of lymphoma.

## References

[bib-001] Cohen J, Pinching A, Rees A, Peters D (1982). A study of the infective complications of 75 patients with immunologically-mediated disease. Q J Medicine.

[bib-002] Bernatsky S, Ramsey-Goldman R, Isenberg D, Rahman A, Dooley M, Sibley J (2007). Hodgkin's lymphoma in systemic lupus erythematosus. Rheumatology.

[bib-003] Bernatsky S, Ramsey-Goldman R, Lachance S, Pineau C, Clarke A (2006). Lymphoma in a patient with systemic lupus erythematosus. Nat Clin Pract Rheumatol.

[bib-004] Drudi FM, Laghi A, Iannicelli E, Di Nardo R, Occhiato R, Poggi R (1997). Tubercular epididymitis and orchitis: US patterns. Eur Radiol.

[bib-005] Muttarak M, Peh W (2006). Tuberculous Epididymo-orchitis. Radiology.

[bib-006] (2000). Subcommittee of the Joint Tuberculosis Committee of the British Thoracic Society: Management of opportunist mycobacterial infections: Joint Tuberculosis Committee Guidelines. Thorax.

[bib-007] Field S, Cowie R (2006). Lung disease due to the more common nontuberculous Mycobacteria. Chest.

[bib-008] Corti M, Palmero D (2008). Mycobacterium avium complex infection in HIV/AIDS patients. Expert Review of Anti-infective Therapy.

[bib-009] Senzaki H, Watanabe H, Ishiguro Y (2001). A case of very rare tuberculosis of the testis [abstract]. Nippon Hinyokika Gakkai Zasshi.

[bib-010] Field S, Cowie R (2003). Treatment of mycobacterium avium-intracellulare complex lung disease with a macrolide, ethambutol, and clofazimine. Chest.

